# Supplementary material for: Analysis of Expression of Programmed Cell Death Ligand 1 (PD-L1) and BRAFV600E Mutation in Thyroid Cancer
Source: Cancers (Basel). 2023 Jun 30;15(13):3449. doi: 10.3390/cancers15133449 (PMC10341229; doi:10.3390/cancers15133449)
Supplement: Supplementary file 1 [file cancers-15-03449-s001.zip › cancers-2388831-supplementary.pdf]

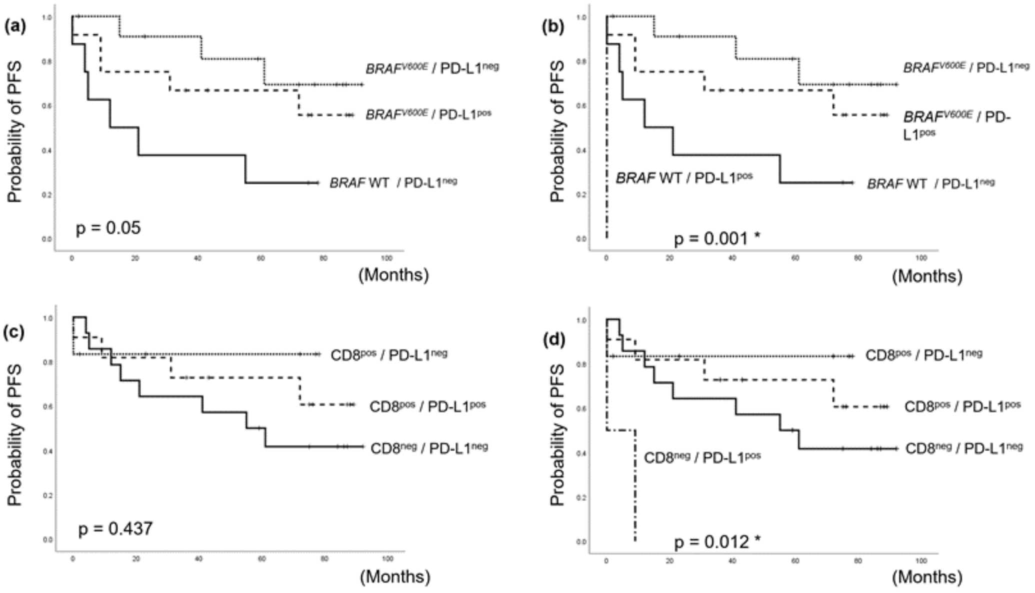

Figure S1. Kaplan-Meier curves for PFS; **(a) (b)** with *BRAF*^{V600E} mutation or wild type and negative or positive PD-L1 expression and **(c) (d)** with negative or positive CD8 + expression and negative or positive PD-L1 expression. P values were calculated by the log- rank test. *BRAF* wild type and PD-L1 positive group (n=1) was excluded from analysis due to small sample. *BRAF*^{V600E}, PD-L1 negative vs. PD-L1 positive; χ^2=0.673, p=0.412. PD-L1 negative, *BRAF*^{V600E} mutation vs. *BRAF* wild type; χ^2=5.615, p=0.018 (a). CD8 + negative/ PD-L1 positive group (n=2) was excluded from analysis. CD8+ positive, PD-L1 negative vs. PD-L1 positive; χ^2=0.317, p=0.573. PD-L1 negative, CD8+ positive vs. CD8+ negative; χ^2=1.140, p=0.286 (c).

Table S1. Results summary

Results	Data details
**Table 1. Correlations between PD-L1 expression and clinicopathological factors in thyroid cancer.**	
1. The present findings indicate the rate of PD-L1 positive expression and clinicopathologic factors which were associated with PD-L1 expression.	With a PD-L1 staining cut-off value of 1%, 13 (39.4%) of 33 patients were classified as positive.
	PD-L1 positive was positively associated with low stimulation Index (SI) levels (p = 0.046).
2. *BRAF*^{V600E} mutation was significantly associated with increasing expression of PD-L1.	12 (50%) of 24 patients with *BRAF*^{V600E} mutation were PD-L1 positive, compared to one (11.1%) of nine patients with *BRAF* wild type (p = 0.047).
3. CD8+ expression was significantly associated with increased PD-L1 expression.	Of the 17 patients with CD8+ positive, 11 (64.7%) were PD-L1 positive, compared to six (35.3%) CD8+ negative patients (p = 0.003).
**Table 2. Univariate analysis of clinicopathological factors associated with PFS.**	
The results of the univariate analysis revealed that 9 variables which were considered significantly associated with poor PFS.<br>PD-L1 expression was not associated with PFS.	9 variables which were considered significantly associated with poor PFS were,<br>・*BRAF* wild type (HR = 0.27 [CI 0.10–0.76], p = 0.013),<br>・high CRP (HR = 5.08 [CI 1.26–64.76], p = 0.002),<br>・high WBC (HR = 30.93 [CI 2.81–340.82], p = 0.005),<br>・high VEGF (HR = 9.02 [CI 1.559–6.383], p = 0.029),<br>・primary tumor size ≥20mm (HR = 7.79 [CI 1.74–34.78], p = 0.007),<br>・pT4 (HR = 8.12 [CI 2.07–31.86], p=0.003),<br>・extrathyroidal extension (Ex) 2 (HR = 9.3 [CI 2.28–37.9], p = 0.002),

	• metastasis (M)1 (HR = 5.73 [CI 1.98–16.58], p = 0.001), and<br>• anaplastic thyroid cancer (HR = 4.97 [CI 1.53–16.18], p = 0.008).
**Table3. Multivariate analysis of clinicopathological factors associated with PFS.**	
In the multivariate analysis, patients with the *BRAF* wild type tended to have poor prognosis than with *BRAF*^{V600E} mutation.	*BRAF* wild type (p = 0.022) and high CRP (p = 0.039) were independent and significant predictive factors for poor PFS.
**Figure 3 and Supplementary Figure S1. Kaplan-Meier estimation of PFS with negative or positive PD-L1 expression, with *BRAF*^{V600E} mutation or wild type, and with negative or positive CD8+ expression.**	
Patients with *BRAF*^{V600E} mutation had a significantly longer survival than with *BRAF* wild type. Moreover, CD8+ negative patients might tend to have a poor prognosis.	*BRAF* wild type patients were significantly associated with poor PFS (p = 0.007).

*SI, Stimulation Index is one of the markers of inflammation; VEGF, vascular endothelial growth factor; BRAF, v-raf murine sarcoma viral oncogene homolog B1; PFS, progression-free survival.
